# Feasibility and acceptability of time-restricted eating in a group of adults with multiple sclerosis

**DOI:** 10.3389/fneur.2022.1087126

**Published:** 2023-01-12

**Authors:** Brooks C. Wingo, John R. Rinker, Kathryn Green, Courtney M. Peterson

**Affiliations:** ^1^Department of Occupational Therapy, University of Alabama at Birmingham, Birmingham, AL, United States; ^2^Department of Neurology, School of Medicine, University of Alabama at Birmingham, Birmingham, AL, United States; ^3^Department of Nutrition Sciences, University of Alabama at Birmingham, Birmingham, AL, United States

**Keywords:** multiple sclerosis, diet, time-restricted eating, intermittent fasting, patient-reported outcomes

## Abstract

**Introduction:**

Intermittent fasting (IF) has become a popular dietary pattern for adults with multiple sclerosis (MS), and initial studies in animal models and human trials indicate promising results for improving symptoms and slowing disease progression. Most studies published to date have focused on alternate day fasting or fasting mimicking diets including a 5:2 pattern, in which participants greatly restrict calorie intake on two non-consecutive days and eat regularly on other days; however, time restricted eating (TRE) may be equally effective for improving symptoms and may lead to better long term adherence due to its focus only on the time of day in which calories are consumed with no restriction on number of calories or types of food consumed.

**Methods:**

The purpose of this pilot study was to determine the feasibility and acceptability of a TRE intervention in adults with relapsing remitting MS (RRMS). Participants (*n* = 12) were instructed to eat all food within an 8-h window every day and fast the remaining 16 h for 8 weeks.

**Results:**

The eating pattern was determined to be feasible based on retention rates (*n* = 11; 92%) and acceptable based on participant feedback.

**Discussion:**

Exploratory results of changes in cognition, pain, and fatigue, indicate that further study of TRE in this population is warranted.

**Clinical trial registration:**

https://clinicaltrials.gov/ct2/show/NCT04389970; NCT04389970.

## 1. Introduction

Over the last two decades, there has been an influx of evidence demonstrating the impact of lifestyle risk factors, including physical inactivity, smoking, and poor diet, on the progression and severity of multiple sclerosis (MS) symptoms. Specifically, epidemiological studies consistently report that a poor diet is associated with an increased risk of disability in adults with MS (1–4). The mechanisms underlying the relationship between diet and MS are not well-understood. A leading theory is that poor diet affects disease progression and symptoms by exacerbating inflammation, either by increasing neuroinflammation directly or by increasing the risk for other pro-inflammatory conditions, including the vascular and metabolic diseases that are common among adults with MS and associated with a worse prognosis of MS.

Intermittent fasting (IF), a dietary pattern characterized by cycles of eating and extended fasting, has shown particular promise for slowing disease progression and reducing symptoms in animal models and early human studies of MS (5–10). There are a number of types of IF, the most popular of which include alternate-day modified fasting (cycling between days of substantially restricted calorie intake and days of unrestricted eating) and time-restricted eating (TRE), in which all food is consumed during a limited daily time window (typically <10 h). While many forms of IF are believed to work primarily by reducing energy intake, evidence demonstrates that TRE may improve health outcomes independent of calorie restriction, and these benefits may at least partially be explained by realigning circadian rhythms (11–13).

Human studies of populations without MS have found that TRE improves insulin sensitivity, 24-h glucose levels, blood pressure, oxidative stress levels, and fat mass (11, 13–17). TRE also has been shown to improve factors that are associated with the onset and progression of MS, such as inflammation, poor immune function, and low levels of neuroprotective agents, including brain-derived neurotropic factor (BDNF), which have all been associated with the onset and progression of MS (14, 15, 18, 19). In addition to its physiological benefits, TRE may also provide a behavioral benefit, as it increases energy levels and improves mood and also addresses barriers to adherence in traditional dietary interventions that explicitly restrict the types and/or quantities of food a participant can eat. In contrast, TRE requires that participants restrict only the timing of their meals (17).

Pilot trials of other forms of IF in adults with MS provide preliminary support for this approach. The first human trials of IF in adults with MS are promising, demonstrating that IF may improve wellbeing (6–8). However, only one previous study has conducted a preliminary investigation of TRE in MS patients (6). The purpose of this pilot study was to determine the feasibility, safety, and acceptability of a TRE intervention and to explore its potential to improve MS clinical outcomes and patient-reported outcomes (PROs) in adults with relapsing remitting MS (RRMS).

## 2. Methods and materials

This prospective single-group pilot study aimed to assess the feasibility of a TRE intervention for adults with RRMS. Specifically, the study sought to determine feasibility of recruitment and retention of the target population, as well as safety and acceptability of the dietary intervention. The study's secondary aim was to explore the preliminary efficacy of an 8-week TRE intervention on MS outcomes.

The Institutional Review Board at the University of Alabama at Birmingham (UAB) approved the study protocol (approval number IRB-300005334), and study investigators obtained written informed consent from all participants prior to baseline data collection. The study was registered with ClinicalTrials.gov (NCT04389970).

### 2.1. Participants

Participants were recruited from a database of previous study participants, as well as direct mail to patients of the UAB MS clinics. Participants met the following criteria: aged 18–65 years; diagnosed with RRMS; if receiving disease-modifying treatment (DMT), stable on current DMT for 6 months; if not receiving DMT, no DMT in the previous 6 months; no MS relapse in the previous 30 days; body mass index (BMI) between 18.5 and 50.0 kg/m^2^; and able to walk 25 feet with or without assistance. Individuals were excluded if they had a score of 31 or greater on the Modified Telephone Interview for Cognitive Status (TICS-m) (20), were actively engaged in a weight loss program, regularly fasted for ≥15 h/day, were pregnant or breastfeeding, or used insulin to control diabetes.

### 2.2. Study diet

Participants were instructed to eat all meals in an 8-h window each day. To maximize adherence, participants were allowed to choose the times at which they ate but had to start their eating window no later than 11 am. During the 16-h fasting period, participants were instructed not to eat any food and to drink only water, unsweetened tea, or black coffee. Participants were instructed not to change the amount or type of food they typically consumed, with a goal of maintaining their baseline weight (i.e., an eucaloric diet based on the Harris-Benedict equation) but did not have any other restriction on food intake.

Throughout the intervention, adherence was self-reported *via* electronic food logs and participant questionnaires. Participants used the HealthWatch 360 mobile app or website (GB HealthWatch, San Diego, CA) to log the type and amount of all food consumed and the time of all food intake. The research portal of HealthWatch 360 allows study staff to track participant entries on a HIPAA-compliant platform; this allowed the study coordinator to review each participant's adherence to TRE prior to a weekly phone call. Participants also reported the number of days a week they ate in the 8-h window on a satisfaction survey administered half-way through and at the end of the study. To measure any changes in food intake, multi-pass 24-h food recalls were conducted at baseline and post-intervention. Participants completed three unannounced telephone recalls at baseline and post-intervention, and values for the three recalls were averaged to determine calorie and macronutrient intake. Recall data was analyzed with NDSR 2020 version (Nutrition Coordinating Center, University of Minnesota, Minneapolis, MN).

The study coordinator called all participants each week of the intervention. During these calls, the coordinator reviewed food records with the participants, collected adverse events, answered questions, and helped those having trouble adhering to TRE problem solve ways to improve adherence.

### 2.3. Measures

#### 2.3.1. Primary outcomes

##### 2.3.1.1. Feasibility

The primary outcome of this study was feasibility, as measured by the feasibility of recruitment, retention, and adherence to TRE. Feasibility of recruitment was measured as the time to enroll the full sample (*n* = 12). Other aspects of the feasibility of recruitment included the number screened to enroll the full sample and the primary reasons for exclusion. Retention was defined as the number of participants completing post-intervention measures. Adherence to the protocol was measured using the number of days each week in which participants adhered to the eating window and using the number of weekly calls they completed with the study coordinator.

##### 2.3.1.2. Participant satisfaction

Participants completed a brief satisfaction survey half-way through and at the end of the study. The survey included rating scales related to difficulties following the TRE intervention, positive and negative changes experienced while following the intervention, mood, hunger, and TRE's interference with social and work-related activities. Scales related to challenges and positive and negative changes were followed by open-ended questions asking participants to provide more information related to their answer choice. The post-intervention satisfaction survey also included questions asking if participants planned to continue to follow the TRE intervention after completing the study.

#### 2.3.2. Secondary outcomes

##### 2.3.2.1. MS outcomes

Clinical outcomes were measured *via* the Multiple Sclerosis Functional Composite (MSFC) (21, 22). The three MSFC components included were the timed 25-foot walk (T25FW), the 9-hole peg test (9HPT), and the symbol digit modalities test (SDMT). The MSFC was conducted at baseline and at the end of the intervention.

##### 2.3.2.2. PROs

Patient-reported disability level was measured *via* the Patient-Determined Disease Steps scale (PDDS) (23). This was only measured at baseline, as results were not expected to change within the short duration of the intervention. Patient-reported outcomes included self-report of pain, mood and anxiety, fatigue, and sleep, which were measured with the short-form McGill Pain Questionnaire (SF-MPQ) (24), the Hospital Anxiety and Depression Scale (HADS) (25), the Modified Fatigue Impact Scale (MFIS) (26) and Fatigue Severity Scale (FSS) (27), and the Pittsburg Sleep Quality Index, respectively (28). All PROs other than PDDS were measured at baseline and at the end of the intervention.

##### 2.3.2.3. Anthropometrics

Height was measured with a wall-mounted stadiometer. Weight was assessed using a digital scale. BMI was calculated using the formula weight/height^2^ (kg/m^2^) (29). Waist circumference was measured twice at the level of the umbilicus and reported as the mean of the two measures. Anthropometrics were measured at baseline and at the end of the intervention.

##### 2.3.2.4. Body composition

Fat and lean mass were assessed by dual energy X-ray absorptiometry (DXA) and analyzed with enCORE software version 13.6 (GE Healthcare, Chicago, IL). Individuals whose body size precluded a single-image scan were scanned twice (one scan each for the left and right halves of the body), and the mean of the two scans was recorded. DXA was conducted at baseline and at the end of the intervention.

### 2.4. Sample size and statistical analysis

The goals of this study were to determine the feasibility of TRE and to collect exploratory pilot data. A sample of *n* = 10–12 per group has been reported to be adequate for a pilot study when there are no existing data available (30, 31). Given the lack of previous data on TRE in adults with MS, a sample size of *n* = 12 was determined to be adequate. An a priori cut point for feasibility was set at 80% of participants completing all pre- and post-intervention measures.

Feasibility variables were assessed with descriptive statistics, specifically frequency and mean and standard deviation. Secondary outcomes were explored descriptively and with single-group paired samples *t*-tests. Significance values are presented, and results are highlighted whenever either the changes were clinically meaningful and/or *p* < 0.20, a standard threshold used for pilot studies; however, due to the exploratory nature of these analyses, the study was not powered to find significance among changes in these variables. Cohen's *d* was calculated to determine the effect size of changes between baseline and follow-up. Given the small sample and exploratory nature of the study, all paired-samples analyses included only those participants who completed the study. All statistical analyses were conducted with SPSS v 27 (IBM Corporation; Armonk, NY).

## 3. Results

Recruitment started in October 2020 and was completed 7 months later in May 2021. Thirty-one individuals were assessed for eligibility. Twenty-five met inclusion criteria, and 12 agreed to participate. The most common reasons for ineligibility were not having a diagnosis of RRMS (*n* = 4) and not meeting DMT criteria or being on insulin (*n* = 3). Twelve participants completed baseline testing and were enrolled in the intervention (Table 1), and 11 (92%) completed the intervention and follow-up measures. The sample enrolled was 83% female and 42% African American. Table 1 describes the full characteristics of the study sample.

**Table 1:** Participant characteristics (*n* = 12).

	***n* (%)**	**Mean (SD)**
**Sex**		
Male	2 (17%)	
Female	10 (82%)	
**Age**		46 (10)
**Age at diagnosis**		36 (10)
**Race**		
White	7 (58%)	
African American	5 (42%)	
**Educational level**		
Some college	2 (16%)	
College/university degree	8 (66%)	
Graduate/professional degree	2 (16%)	
**Income level**		
$35,000–74,999/yr	5 (42%)	
>$75,000/yr	3 (25%)	
No response	4 (33%)	
**Marital status**		
Married	5 (42%)	
Never married	3 (25%)	
Separated	1 (8%)	
Divorced	3 (25%)	
**Number of people in household**		
1	4 (33%)	
2	3 (25%)	
3	1 (8%)	
≥ 4	4 (33%)	
**Assistive device (used at any time, not necessarily primary mode of ambulation)**		
None	9 (75%)	
Cane	3 (25%)	
**PDDS**		
Normal function (0)	6 (50%)	
Minimal gait disability (1)	2 (17%)	
Mild gait disability (2)	2 (17%)	
Occasional use of cane/unilateral support (3)	1 (8%)	
Frequent use of cane (4)	0 (0%)	
Severe gait disability/bilateral support (5)	1 (8%)	
Total gait disability (6)	0 (%)	

PDDS, patient determined disease steps.

### 3.1. Intervention adherence

Participants reported eating within the 8-h window a mean (SD) of 6.8 (1.6) days/week in the middle and 6.5 (1.4) days/week at the end of the 8-week intervention. They completed 87% of scheduled calls with the study coordinator and recorded at least one meal in the HealthWatch food journal on 46% of intervention days [mean (SD) = 27 (22) days]. Based on 24-h recalls, participants did not change their daily energy or macronutrient intake during the intervention (Table 2).

**Table 2:** Daily diet intake (completers only).

	**Baseline mean (SD)**	**Follow-up mean (SD)**	***p***	**Paired difference (SD)**	**Cohen's *d***
Calories	1,434.5 (393.9)	1,377.0 (494.5)	0.37	57.5 (193.23)	0.30
Percent of calories from protein	17.6 (6.2)	19.0 (7.0)	0.30	−1.4 (4.0)	0.35
Percent of calories from fat	38.2 (4.7)	38.2 (8.7)	0.99	−0.03 (10.1)	0.003
Percent of calories from carbohydrate	41.1 (7.1)	40 0(11.7)	0.70	1.1 (8.7)	0.50

### 3.2. Participant perspective

All study completers and 73% of study completers (*n* = 8) completed satisfaction surveys at midpoint and endpoint, respectively. Two participants noted sleeping better as a positive benefit of TRE. The following were additional benefits noted by one participant each: having more energy, feeling better overall, reduced acid reflux, weight loss, being more mindful of what they ate, and drinking more water. Two participants noted feeling that they had to schedule their social life, working hours, and family schedule around their eating window as a negative effect of TRE. Constipation, headache, and weight gain were each noted by one participant. At endpoint, *n* = 4 (50%) of responders indicated that they planned to continue TRE and noted they would make some adjustment to their meal schedule to better fit their lifestyle and schedule. These adjustments included shifting the eating window earlier or later to accommodate work and social schedules and reducing days following TRE to 5–6 days/week to allow for more flexibility.

### 3.3. Adverse events

No serious adverse events were reported. Headaches and constipation were reported by one participant each.

### 3.4. Secondary outcomes

**Figure 1:**
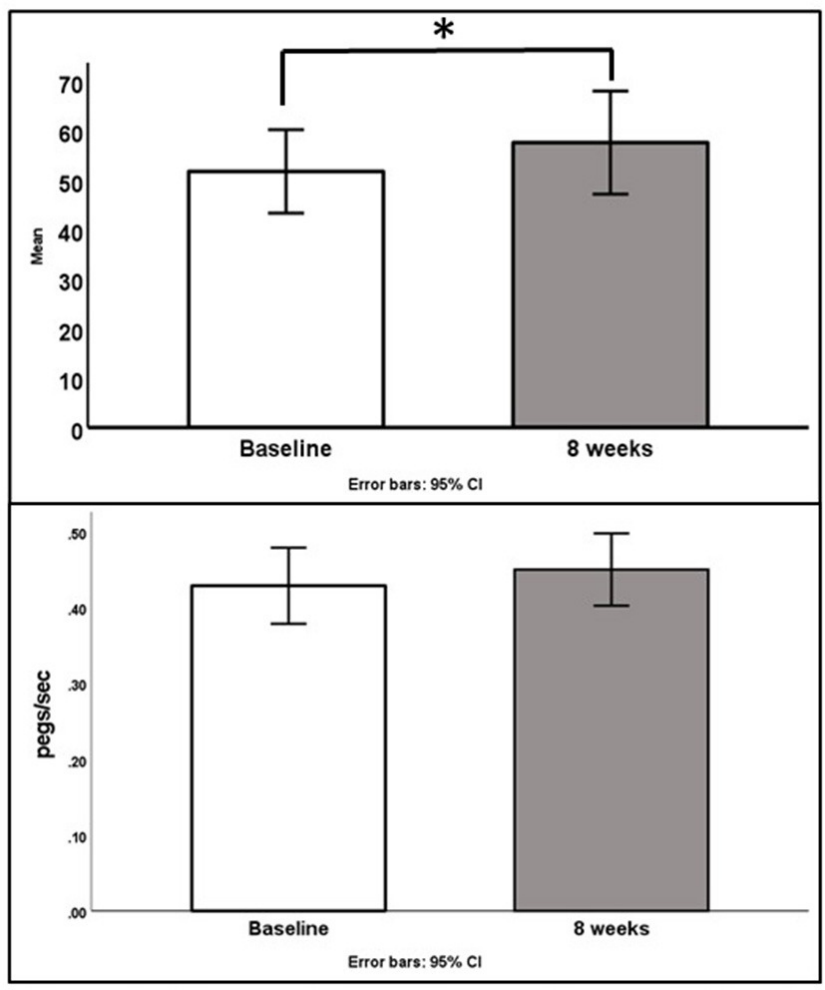
Changes in SDMT scores (**top**, *p* = 0.04) and 9 hole peg test speed (**bottom**, *p* = 0.06).

Changes to MSFC and PROs are shown in Table 3. Symbol digit modalities scores and speed on the 9-hole peg test improved by 5.8 (8.3) (*p* = 0.04) and 0.02 (0.03) ft/sec (*p* = 0.06; Figure 1) respectively. Speeds on the 25-foot walk test did not appreciably change. Total pain scores decreased by 1.9 [5.4] (*p* = 0.3). Fatigue scores on the MFIS indicated there may be a slight improvement in psychosocial fatigue [−0.5 (1.6); *p* = 0.29]. However, fatigue measured by the FSS did not improve. No clinically meaningful differences were found in sleep, mood, or anxiety scores. Participants maintained their baseline weight (*p* = 0.69) and body composition at the end of the intervention.

**Table 3:** Patient reported outcomes, clinical outcomes and cardiometabolic risks (completers only).

	**Baseline mean (SD)**	**Follow-up mean (SD)**	***p***	**Paired difference (SD)**	**Cohen's *d***
Timed 25-ft walk (ft/sec)	4.9 (1.4)	4.6 (1.4)	0.39	−0.3 (1.2)	0.27
9-hole peg test (peg/sec)	0.43 (0.07)	0.45 (0.07)	0.06	0.02 (0.03)	0.64
Symbol digit modalities test	51.7 (12.5)	57.6 (15.5)	0.04	5.8 (8.3)	0.70
Sleep (PSQI global score)	8.6 (3.0)	8.8 (4.2)	0.88	0.2 (4.0)	0.05
Fatigue (FSS)	30.5 (14.0)	32.8 (13.2)	0.66	2.3 (16.7)	0.14
Fatigue (MFIS total score)	33.5 (15.2)	32.2 (17.2)	0.77	−1.3 (14.3)	0.09
Physical subscale	15.1 (6.7)	14.3 (7.7)	0.64	−0.8 (5.7)	0.14
Cognitive subscale	15.3 (7.1)	15.5 (8.4)	0.97	0.1 (7.8)	0.01
Psychosocial subscale	3.0 (2.1)	2.5 (1.7)	0.29	−0.5 (1.6)	0.33
Mood	4.1 (2.6)	3.9 (3.6)	0.83	−0.2 (2.8)	0.06
Anxiety	6.9 (3.0)	7.2 (4.2)	0.82	0.3 (3.9)	0.07
Pain (McGill total score)	9.9 (10.4)	8.0 (11.3)	0.30	−1.9 (5.4)	0.35
Sensory subscale	7.5 (7.6)	6.3 (8.1)	0.38	−1.2 (4.9)	0.28
Affective subscale	1.7 (2.9)	1.7 (2.8)	1.0	0.0 (1.9)	0.00
Weight (kg)	79.9 (9.1)	80.3 (9.6)	0.12	0.4 (3.3)	0.05
BMI (kg/m^2^)	28.7 (3.3)	29.1 (3.3)	0.35	0.4 (1.1)	0.35
Waist circumference (cm)	91.9 (8.2)	92.0 (8.8)	0.02	0.1 (3.7)	0.16
Lean mass (% of total body mass)	58.2 (8.2)	58.2 (7.9)	0.03	0.0 (1.0)	03
Fat mass (% of total body mass)	39.9 (8.6)	39.9 (8.2)	0	0.00 (1.0)	0.00

BMI, body mass index; FSS, Fatigue Severity Scale; MFIS, Modified Fatigue Impact Scale; PSQI, Pittsburgh Sleep Quality Index.

## 4. Discussion

The primary purpose of this study was to determine the feasibility and acceptability of TRE in adults with RRMS. Results indicate that TRE may be feasible and acceptable in adults with MS, as evidenced by feasible recruitment, high retention, high levels of adherence, and positive participant feedback. Participants adhered to TRE about >6.5 days/week throughout the trial, and only one participant dropped out of the intervention. Further, participants reported qualitative improvements in fatigue, sleep, and wellbeing.

However, the 8-h TRE window we tested may need to be further refined to address MS-specific symptoms and medication management to promote optimal adherence in this population. Specifically, the requirement to begin the eating window no later than 11:00 am may have posed a challenge for some MS patients. We required the eating window to be not too late in the day due to a growing body of evidence suggesting that eating earlier in the day is more beneficial for metabolic health than eating later in the day (11, 12). However, some participants noted their sleep schedules made starting the eating window by 11 am difficult. Given that fatigue is one of the most common symptoms of MS, individuals who sleep later may find it difficult to start eating in the morning. Future research should explore the interaction of sleep schedules and meal timing in adults with MS.

The secondary goal of this study was to explore the potential for TRE to improve MS clinical outcomes. Despite the fact that this study was not powered to detect significant changes in MS outcomes, some markers indicated change in the anticipated direction. Specifically, TRE reduced fatigue and pain and improved cognition by clinically significant amounts. Scores on the sensory subscale of the McGill Pain Questionnaire were the most notable change in pain. Intermittent fasting is believed to impact pain specifically through reduction in inflammatory cytokines and oxidative stress (32). It may also increase synaptic plasticity and aid in preservation of myelin (32, 33). Reductions in fatigue were most pronounced in the physical and psychosocial subscales of Modified Fatigue Impact Scale. Studies of TRE in healthy adults and samples with obesity demonstrate improved fatigue and mood (17), and the first study in adults post cancer recently reported decreases in fatigue after 2 weeks of TRE (34). Although other dietary interventions including calorie restriction have shown improvements in fatigue among adults with MS (35), these are the first data to hint that TRE could possibly reduce fatigue and pain and improve symptoms of MS.

While there is evidence that IF improves cognition in a small number of animal models, very few human trials have explored the impact of IF on cognition, and no reports of TRE have been published. Animal studies indicate that IF can impact the brain in several ways, including by reducing inflammation, activating autophagy, and synchronizing peripheral and central circadian rhythms (36). Animal models of neurological conditions including ischemic stroke and Alzheimer's disease suggest that fasting interventions reduce cognitive decline, spatial memory deficits, and hippocampal damage in animals (37). It is important to note, however, that due to the limited amount of research on IF and the theorized mechanisms, many IF studies involve calorie restriction, so some of the results may not translate into humans who follow a weight-stable TRE intervention like the one used in the current trial. There are only two known reports of IF on cognition in humans. Ooi et al. reported enhanced cognitive function in adults with mild cognitive impairment following 3 years of Sunnah fasting (sunrise to sunset, Monday-Thursday) when compared to non-fasters (38), while Kim reported no additional benefits for cognition in adults with central obesity who followed a 5:2 IF intervention for 4 weeks when compared to those on daily calorie restriction (39). The present study is the first known report of the potential impact of TRE on cognition in adults with MS, and additional study is warranted.

A limitation of this study is its measurement of dietary adherence. Although participants self-reported they adhered ≥6.5 days half-way through and during the last week of the study, mealtimes on the 3-day 24-h food recalls suggest they overestimated their adherence. In recalls, only 60% conformed to the 8-h window on at least one of the 3 days reported. In addition, many participants did not enter the foods they ate in the electronic food journal as instructed, in part because they were monitoring only when they ate rather than what they ate. This precluded the review of daily intake time records. This suggests that the burden of journaling food intake on a daily basis may have been too much burden for MS patients. Although these methods for monitoring adherence align with reported methodology from some previous TRE studies, future studies need to find more precise ways of monitoring meal timing to produce more accurate data on adherence.

We acknowledge that the sample size of this study was too small to allow for adequate significance testing; however, the primary goal of this pilot study was to determine intervention feasibility and to calculate effect sizes to inform power calculations of large future trials. Additionally, as a single-arm trial it is not possible to determine if participants in this study would have similar outcomes with a different diet, or with no change in diet. Additionally, the study's short duration may not have been sufficient to affect some of the variables measured. Our future studies will focus on completing longer interventions with an appropriate control group and a time frame adequate for full assessment of the efficacy of TRE for people living with MS.

## 5. Conclusion

TRE may be a low-cost, highly scalable dietary intervention for adults with RRMS. Our data suggest that it is worth testing the hypothesis that TRE reduces pain and fatigue and improves cognition in a large-scale trial in people with MS. TRE has behavioral advantages of flexibility and focusing only on meal timing, rather than quantity or type of food, which may improve long-term adherence compared to traditional dietary prescriptions based on calorie or macronutrient restriction. While the sample size and study design of the current study cannot be interpreted as causal, our promising preliminary findings warrant additional research that focuses on using TRE to reduce MS symptoms.

## Data availability statement

The datasets presented in this article are not readily available because of ethical and privacy restrictions. Requests to access the datasets should be directed to BW, bcwingo@uab.edu.

## Ethics statement

The studies involving human participants were reviewed and approved by University of Alabama at Birmingham. The patients/participants provided their written informed consent to participate in this study. Written informed consent was obtained from the individual(s) for the publication of any potentially identifiable images or data included in this article.

## Author contributions

BW conceived and designed the study, oversaw data collection, analyzed data, and wrote the paper. CP assisted with study design, data interpretation, and writing the paper. JR assisted with study design, served as medical oversight for the study, and assisted with data interpretation and writing the paper. KG served as study coordinator and edited the paper. All authors contributed to the article and approved the submitted version.
